# Efficacy and Mechanism of Preoperative Simvastatin Therapy on Myocardial Protection after Extracorporeal Circulation

**DOI:** 10.1155/2017/6082430

**Published:** 2017-11-08

**Authors:** Ping Hua, Jianyang Liu, Jun Tao, Rongjun Zou, Xifeng Lin, Dingwen Zhang, Songran Yang

**Affiliations:** ^1^Department of Cardiovascular Surgery, Sun Yat-sen Memorial Hospital, Sun Yat-sen University, Guangzhou 510120, China; ^2^Department of Vascular Surgery, Henan Provincial People's Hospital, Zhengzhou 450003, China; ^3^The Biobank of Sun Yat-sen Memorial Hospital, Sun Yat-sen University, Guangzhou 510120, China; ^4^Guangdong Province Key Laboratory of Brain Function and Disease, Zhongshan School of Medicine, Sun Yat-sen University, Guangzhou 510080, China

## Abstract

**Background:**

Cardiopulmonary bypass (CPB) causes systemic inflammatory response and ischemia-reperfusion (IR) injury.

**Objective:**

To investigate the effect and mechanism of simvastatin on myocardial injury in cardiac valve surgery with CPB.

**Methods:**

One hundred thirty patients were randomly assigned to the statin group (*n* = 65) or control group (*n* = 65). Simvastatin was administered preoperatively and postoperatively. Duration of intensive care unit stay, duration of assisted ventilation, and left ventricular ejection fraction were recorded. Plasma was analysed for troponin T (cTnT), isoenzyme of creatine kinase (CK-MB), tumor necrosis factor alpha (TNF-*α*), interleukin-6 (IL-6), and interleukin-8 (IL-8). Ultrastructure of the myocardium and autophagosomes were observed. Beclin-1, LC3-II/I, P62, AMPK, and the phosphorylation of AMPK in cardiomyocytes were detected.

**Results:**

Simvastatin significantly reduced the duration of assisted ventilation (*P* = 0.030) and ejection fraction was significantly higher in the statin group (*P* = 0.024). Simvastatin significantly reduced the levels of cTnT, CK-MB, TNF-*α*, IL-6, and IL-8 (*P* < 0.05), reduced the expression of LC3-II/LC3-I and Beclin 1, and increased the expression of phosphorylation of AMPK. Simvastatin reduced the generation of autophagosomes and the ultrastructural injuries to myocardium.

**Conclusion:**

Perioperative statin therapy reduced myocardial injury by regulating myocardial autophagy and activating the phosphorylation of AMPK. The registration number of this study is ChiCTR-TRC-14005164.

## 1. Introduction

Cardiopulmonary bypass (CPB) is used in most open-heart surgeries. However, CPB inevitably causes systemic inflammatory response and ischemia-reperfusion (IR) injury, which can negatively affect postoperative cardiac function and a patient's long-term prognosis [1].

Recent clinical studies have reported that using statins during the perioperative period can limit inflammation and oxidation, reduce cardiac muscle injury, and improve the patient's prognosis [2–4], but the dosage, duration, effects, and mechanisms associated with these outcomes are not clear. Some studies have demonstrated that statins can induce cell autophagy [5]. IR injury and inflammatory response can substantially activate cell autophagy [6], and autophagy has been demonstrated to play an important role in the pathologic process of cardiac muscle injury [7]. However, few studies have revealed the effects of perioperative oral simvastatin on post-CPB changes in cardiac muscle autophagy or the influence of the degree of autophagy on postoperative cardiac function and prognosis [8, 9].

The present clinical randomized controlled trial explored the protective effect of perioperative simvastatin on cardiac muscle after CPB and the relationship of such effects to cardiomyocyte autophagy.

## 2. Methods

### 2.1. Patients

The study was approved by the Ethics Committee of Sun Yat-sen Memorial Hospital, Sun Yat-sen University. To estimate the group size, a pilot study was conducted for measuring the serum cardiac troponin T (cTnT) at 6 h after surgery in 10 patients who received statin and placebo treatment (ratio 1 : 1). The mean of the cTnT in statin and placebo groups was 830 and 995, respectively. For our sample size calculation, we assumed an equal standard deviation which was 300 at 6 h after surgery among the groups. With *α* = 00.05, two-tailed and a power of 80%, we needed 52 patients per group. Considering a compliance rate of 80%, we asked 130 patients to participate in this study [10]. Included were 130 patients with valvulopathy who underwent surgery with CPB in the department of cardiac surgery at our hospital from February 2013 to December 2014. Patients provided written informed consent and were randomly divided into 2 groups (65 in the simvastatin group and 65 in the control group) by assigning random numbers from a random number table.

Patients in the simvastatin group took simvastatin 20 mg orally daily during two perioperative periods: from the 7th to the 5th preoperative day and from the 2nd day after decannulation until the 7th postoperative day. Patients in the control group were given a placebo at the same dosage.

Patients in the simvastatin group took simvastatin 20 mg orally daily during two perioperative periods: those who received simvastatin for 5~7 days before the planned operation [11] and from the 2nd day after decannulation until the 7th postoperative day.

Exclusion criteria were the following: (1) coronary heart disease; (2) allergy to simvastatin; (3) active hepatitis and/or severe renal impairment (serum creatinine > 200 *μ*mol/L); (4) pregnancy or lactation; (5) taking medication(s) that may increase the effective concentration of simvastatin (e.g., a tetralol-derived calcium-channel blocker such as mibefradil); and (6) hyperlipidemia.

Patients who needed to stop or reduce the dosage of simvastatin because of side effects, such as transaminase elevation, were removed from the study. A patient could voluntarily exit the study at any time, and reason(s) for exiting and data collected to that point were recorded. Women of childbearing age were required to use contraception; however, simvastatin was immediately discontinued if pregnancy occurred.

### 2.2. Anesthetic and Operative Methods

Anesthesia was induced with midazolam 0.1 mg/kg, propofol 0.3–0.5 mg/kg, fentanyl 3–5 *μ*g/kg, and rocuronium bromide 0.6 mg/kg and was maintained with sevoflurane, fentanyl, and rocuronium bromide.

All patients were managed on by the same operating team. A mid-sternal incision was made and, after systemic heparinization, CPB with mild hypothermia was applied using a Stockert extracorporeal circulation machine and membrane oxygenator. Lactated Ringer's solution with 5% human serum albumin was used as a priming solution. Perfusion was maintained at 50–70 mL·kg^−1^·min^−1^, and mean arterial pressure was maintained at 50–80 mm Hg. Ice was put on the surface of the heart to reduce the temperature. After clamping the aorta, cold oxygenated-blood potassium-containing cardioplegia was perfused into the root of the ascending aorta. If the aorta was clamped for longer than 30 min, cardioplegic solution was reperfused to protect cardiac muscle. After the circulation was opened and the heart resuscitated, a pulmonary artery catheter was inserted into the right ventricle and a temporary epicardial pacemaker wire was placed on the heart's surface. After the surgery, the patient was transferred, intubated, to the cardiac surgery intensive care unit.

### 2.3. Clinical Data and Sample Collection

Data recorded were age, weight, duration of CPB, duration of aortic clamping, 24 h postoperative urine output and volume of thoracic cavity drainage, duration of use of vasoactive agents, and duration of intensive care unit stay.

A 5 mL blood sample was taken from a central vein 6, 12, 24, and 72 h and 7 d pre- and postoperatively. Each sample was placed in an anticoagulative tube or treated with heparin before hypothermal ultracentrifugation to separate serum for measurement. Measurements included inflammatory indices (tumor necrosis factor alpha [TNF-*α*], interleukin-6 [IL-6], and interleukin-8 [IL-8]) 6 h pre- and postoperatively, and cardiac muscle impairment indices (serum cardiac troponin T [cTnT] and creatine kinase myocardial-band isoenzyme [CK-MB]) 6, 12, 24, and 72 h pre- and postoperatively.

### 2.4. Tissue Sample Indicator Measurement

Approximately 100 mg of auricula extra tissues was sampled from each patient before CPB and 30 min after opening the aorta. About 50 mg was quick-frozen in liquid nitrogen and then stored in a −80°C freezer for Western blotting measurement of the autophagy-related proteins Beclin-1, LC3-II/I, P62, AMPK, and p-AMPK. The remaining 50 mg was fixed in 4% paraformaldehyde solution, embedded in paraffin, and sliced vertically into 4 to 6 *μ*m thick slices for immunohistochemical (IHC) detection of Beclin-1 and P62. In addition, 1 mm^3^ of the sample was excised and fixed in 2.5% glutaraldehyde for observation under a transmission electron microscope (TEM).

## 3. Statistical Analysis

SPSS for Windows, Version 13.0 (SPSS Inc., Chicago, IL, USA) was used for statistical analysis. All numeric values were expressed as mean ± standard deviation (SD). Student's *t*-test was used for intragroup comparison. ANOVA for repeated measurement was used for intergroup comparison, and numeric values were verified using the chi-squared test. Differences with a *P* value < 0.05 were considered statistically significant.

## 4. Results

### 4.1. Simvastatin Significantly Improves Perioperative Mechanical Ventilation and Cardiac Function

One hundred thirty patients were randomly assigned to either the simvastatin group or the control group; 110 patients completed the study (56 in the simvastatin group and 54 in the control group). There were no significant differences in clinical indicators (age; sex; height; weight; cardiac function rating; preoperative ejection fraction; blood lipid levels before taking, and after stopping, the medication [or placebo]; duration of CPB; and duration of aortic clamping between groups) (Tables 1 and 2).

Nine patients in the simvastatin group left the study for the following reasons: two developed severe liver function abnormality after surgery, five failed to take the medication for the specified number of days, one underwent repeat thoracotomy, and one died while staying in the ICU. In the control group, eleven patients left the study: two developed severe postoperative liver function abnormality, five failed to take the medication for the specified number of days, two exited the study voluntarily, one underwent repeat thoracotomy, and one died while staying in the ICU.

There were no significant differences in 24 h urine output, 24 h volume of thoracic cavity drainage, and duration of ICU stay between groups. The duration of mechanical ventilation was significantly shorter in the simvastatin group (*P* = 0.030). By the 10th postoperative day, left ventricular ejection fraction had decreased in both groups but was significantly smaller in the control group (*P* = 0.024). In both groups, serum lipid concentrations were smaller than before medication administration, but there were no significant differences between groups (Table 3).

### 4.2. Simvastatin Significantly Reduces the Levels of Myocardial Injury Indicators

**Figure 1:**
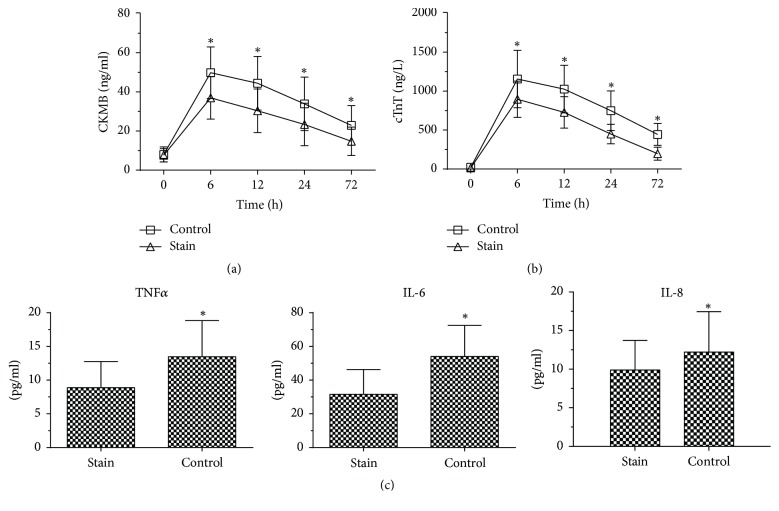
At all postoperative time points, the indices of cardiac muscle injury, CK-MB and cTnT, were significantly lower in the simvastatin group than in the control group. ^*∗*^*P* < 0.05 versus control group. Net increases in serum IL-6, IL-8, and TNF-*α* in the simvastatin group and control group between 6 h before and 6 h after operation.

Preoperatively, indices of cardiac muscle injury, serum CK-MB and cTnT, were similar between groups and low levels. Postoperatively, serum CK-MB and cTnT both increased dramatically, reaching peak concentration 6 h after the operation and then gradually decreasing. Serum CK-MB and cTnT were significantly lower in the simvastatin group than in the control group at all time points after the operation (Figure 1).

### 4.3. Simvastatin Significantly Reduces the Levels of Inflammatory Markers after CPB

Blood inflammatory cytokines were similar between groups preoperatively. However, 6 h after CPB there were clear increases in all three inflammatory markers in both groups, with the increases significantly smaller in the simvastatin group than in the control group for IL-6, IL-8, and TNF-*α* (*P* < 0.05) (Figure 1).

### 4.4. Simvastatin Significantly Reduces the Levels of Postoperative Myocardial Autophagy

**Figure 2:**
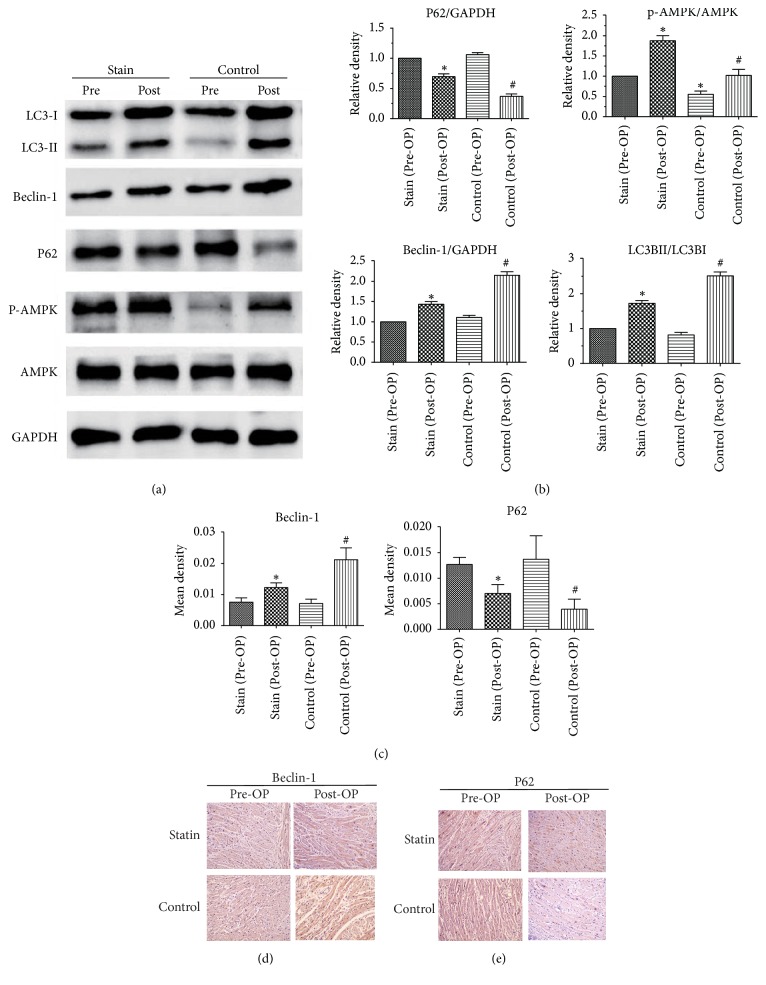
((a), (b), and (c)) Expression of LC3-II, Beclin-1, P62, and p-AMPK by Western blotting assays in both groups before and after surgery. Data expressed as mean ± SD. *n* = 62–64. ^*∗*^*P* < 0.05 versus statin (Pre-OP); ^#^*P* < 0.05 versus statin (Post-OP). ((d) and (e)) Expression of Beclin-1 and P62 by immunohistochemistry in both groups before and after surgery. Magnification 200x. Data expressed as mean ± SD, *n* = 62–64. ^*∗*^*P* < 0.05 versus statin (Pre-OP); ^#^*P* < 0.05 versus statin (Post-OP).

Western blotting assays showed that, after ischemia reperfusion during CPB, cardiomyocyte autophagy was activated in both groups. Although Beclin-1, LC3-II, and LC3-II/LC3-I ratios increased in both groups, increase was significantly greater in the control group than in the simvastatin group (*P* < 0.05). P62 decreased in both groups but the decrease was significantly higher in the control group than in the simvastatin group (*P* < 0.05). This indicates that activation of autophagy after CPB was suppressed to some extent in the simvastatin group. Total protein content of AMPK was roughly equal between groups, but the degree of phosphorylation of AMPK was significantly greater in the simvastatin group than in the control group (*P* < 0.05), demonstrating that simvastatin activated the phosphorylation of AMPK (Figure 2).

IHC revealed the expression of autophagy-related proteins (i.e., Beclin-1 and P62) and that, in both groups, the expression of Beclin-1 protein increased and that of P62 protein decreased after ischemia reperfusion during CPB, suggesting the activation of cardiomyocyte autophagy. However, results also showed that the expression of autophagy-related proteins was significantly higher in the control group than in the simvastatin group, confirming that simvastatin can suppress further activation of autophagy after CPB (Figure 2).

**Figure 3:**
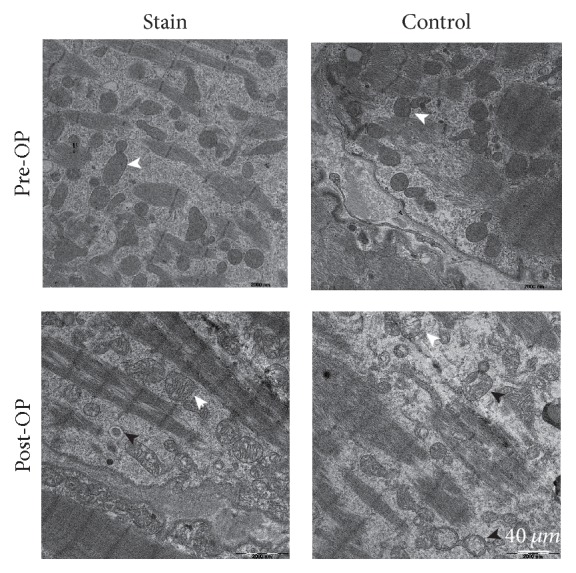
Transmission electron microscopy of myocardial ultrastructure (black arrow indicates autophagosome or autolysosome; white arrow indicates mitochondrion. Magnification 2500x).

Preoperatively, cardiomyocytes were observed under TEM to contain large numbers of mitochondria with a dense matrix, neatly aligned cristae, and enormous glycogen granules. After operation, it could be seen that the cardiomyocyte was seriously injured, with most mitochondria having a swollen matrix and cristae that were shortened, shrunken, or absent. Also, the number of organelles, including glycogen granules and mitochondria, greatly decreased, with a large number of autophagic bodies or autophagic lysosomes at the different stages. Simvastatin can significantly reduce the levels of postoperative myocardial autophagy (Figure 3).

## 5. Discussion

TNF-*α*, the initial factor in an inflammatory response [12], plays a key role in inflammatory response because it can stimulate the release of other inflammatory factors. IL-6 and IL-8 are important cell factors that drive inflammatory response and tissue injury [13]. TNF-*α*, IL-6, and IL-8 concentrations usually peak in the first 6 h after surgery and can reflect the level of post-CPB inflammatory response and change [14]. Our study results show that, although IL-6, IL-8, and TNF-*α* (and, thus, inflammatory response) increased significantly for 6 h postoperatively in both groups, the increase was significantly lower in the simvastatin group. From our results, it can be seen that oral administration of simvastatin can effectively suppress the occurrence and progress of post-CPB inflammatory response.

CK-MB and cTnT are indicators that are clinically used to reflect cardiac muscle injury; they have high specificity and sensitivity and correlate well with the degree of injury [15]. The greater concentrations of CK-MB and cTnT after CPB directly reflect cardiac muscle injury in both groups, but these were less severe in the simvastatin group.

Our clinical results also showed that taking simvastatin during the perioperative period can noticeably shorten the duration of mechanical ventilation and improve left ventricular ejection fraction postoperatively. This translated to moderate improvement in the short-term prognosis of the simvastatin group. Our results also demonstrate that taking a statin during the perioperative period can provide some protective effect to cardiac muscle in patients undergoing cardiac valve replacement. Moreover, no blood lipid abnormalities were seen in either group preoperatively and there was no significant change in blood lipids postoperatively, suggesting that the protection provided by statins during the perioperative period is independent of their ability to reduce blood lipids.

Cardiomyocytes are nonregenerative cells and must therefore maintain a low level of autophagy to remove damaged intracellular structures, which are important for the regulation of cardiomyocyte impairment and aging [16]. In CPB-induced stress states, such as oxidative stress, endoplasmic reticulum stress, mitochondrial impairment, and inflammatory response [17, 18], cardiomyocyte autophagy can be strongly activated [19]. A number of autophagies can eventually lead to autophagic death of the cardiomyocyte, which has been shown to be a probable key cause of the programmed death of cardiomyocytes under stress states [6]. In a study by Matsui et al. [6], Beclin-1^+/−^ hybridized mice showed lower levels of cardiomyocyte autophagy and a smaller area of infarct during reperfusion with decreasing Beclin-1 level. Pattingre et al. proposed that, regardless of whether autophagy leads to the death of a cell, it usually depends on the balance between Beclin-1 and B-cell lymphoma-2 (Bcl-2) genes, which can suppress each other [20]. Bcl-2 can suppress death caused by reactive oxygen species [21], while Beclin-1 has a structural domain that prompts apoptosis [22, 23]. Thus, large activation of Beclin-l during reperfusion suppresses the expression of Bcl-2 and eventually causes the cell's autophagic death. These studies results [16, 23] indicate that suppressing autophagy can reduce cardiac muscle injury.

Our Western blotting assay results indicated that, after CPB, the autophagy-related proteins in the patient's auricula dextra tissue, such as LC3-II/LC3-I and Beclin-1, increased while P62 decreased, which is a clear sign that cardiomyocyte autophagy was activated. At the same time, we found that the autophagy level for simvastatin group was noticeably lower than that of the control group, indicating that simvastatin suppressed cardiomyocyte autophagy after CPB. This was further confirmed by our IHC detection results. By observing the microstructure of cardiomyocytes with TEM, we also found that the cardiomyocytes of patients in the control group were more severely injured, with a swollen matrix; broken or absent mitochondrial cristae; significantly decreased number of organelles, including mitochondria; and significantly increased number of autophagic vacuoles, than those of the simvastatin group. From this, we infer that a great deal of cardiomyocyte autophagy was activated after CPB and that the level of autophagy was positively correlated with the extent of cardiac muscle injury. However, simvastatin may have mitigated cardiac muscle injury by suppressing cardiomyocyte autophagy.

A previous study concluded that statins reduce cell apoptosis mainly by activating AMPK signal path under conditions of* in vitro *hypoxia and a serum-free system [24]. In the present study, we detected the phosphorylation level of AMPK in cardiac muscle tissue and found that the level of phosphorylation of AMPK was higher in the simvastatin group than in the control group. The AMPK signal path is important in adjusting the body's energy metabolism and has been demonstrated to play a key role in IR injury of cardiac muscle. For example, in the event of ischemia and hypoxia, it can optimize cardiac muscle energy metabolism by increasing the ingestion of glucose and oxidation of aliphatic acid and suppressing the synthesis of proteins and consumption of ATP [25]. The AMPK signal path can also resist oxidative stress and suppress endoplasmic reticulum stress while reducing inflammatory factor TNF-*α* to suppress inflammatory response [26, 27]. Oxidative stress, endoplasmic reticulum stress, and inflammatory response are the main causes of induction of cardiomyocyte autophagy in ischemia reperfusion; therefore, we believe that simvastatin may be able to suppress cardiac muscle autophagy and protect cardiac muscle by activating AMPK.

In summary, our results demonstrate that oral administration of simvastatin during the perioperative period effectively mitigated injury caused by CPB, possibly by suppressing cardiac muscle autophagy, and consequently improves the prognosis of patients.

## 6. Limitations

This study has several limitations. First, this study was a short-term follow-up study of the efficacy and mechanism of preoperative simvastatin therapy. Unfortunately, there was a lack in partial follow-up information and we failed to assess the long-term prognosis. Second, the changes of autophagy protein levels in cardiomyocytes after cardiopulmonary bypass were found; however, the changes of autophagy-related upstream signaling pathways were not further studied. Third, the present study lacks the data of postoperative indicators of atrial fibrillation and renal insufficiency.

## Figures and Tables

**Table 1:** Baseline characteristics and comorbidities.

	Simvastatin (*n* = 56)	Control (*n* = 54)	*P* value

Gender (male/female)	20/36	22/32	
Age (year)	56.1 ± 11.1	51.4 ± 12.9	*0.131*
Weight (kg)	56.8 ± 7.9	58.6 ± 8.7	*0.245*
Height (cm)	161.4 ± 6.3	163.4 ± 5.9	*0.094*
CPB duration (min)	115.4 ± 28.7	110.8 ± 36.8	*0.464*
Aorta clamping duration (min)	79.2 ± 22.26	73.9 ± 29.6	*0.296*
Atrial fibrillation (persons)	14	12	*0.847*
Diabetes (persons)	2	3	*0.742*
Ejection fraction EF (%)	60.32 ± 14.97	60.2 ± 6.61	*0.958*
Cardiac function (NYHA class)			*0.704*
I	3	4	
II	14	15	
III	38	33	
IV	1	2	
Blood lipid level			
Total cholesterol (mmol/L)	4.56 ± 0.90	4.79 ± 0.84	*0.171*
Triglyceride **(**mmol/L)	1.15 ± 0.32	1.26 ± 0.29	*0.056*
Low density lipoprotein (mmol/L)	2.65 ± 0.56	2.78 ± 0.48	*0.187*
High density lipoprotein **(**mmol/L)	1.21 ± 0.24	1.24 ± 0.31	*0.469*

Data expressed as mean ± standard deviation. CPB: cardiopulmonary bypass; NYHA: New York Heart Association.

**Table 2:** Details of surgical procedures.

Operation	Simvastatin group (56)	Control group (54)

AVR	9	9
MVR	20	24
MVP	3	4
AVR + MVR	6	3
AVR + MVP	1	0
MVR + TVP	12	7
MVR + TVR	1	2
MVP + TVP	1	1
MVP + TVR	0	1
AVR + MVR + TVP	3	3

*AVR: *aortic valve replacement; *MVP: *mitral valve plasty; *MVR: *mitral valve replacement; *TVP: *tricuspid valve plasty; *TVR: *tricuspid valve replacement.

**(a):** 

Group	Duration of assisted respiration	24 Hr average urine volume	24 Hr total drainage	Duration of staying in ICU	EF 10 days after operation
	(h)	(ml/h)	(ml)	(h)	(%)
Simvastatin group	9.92 ± 3.42^*∗*^	127.73 ± 41.02	770.18 ± 244.79	40.18 ± 13.19	58.9 ± 8.48^*∗*^
Control group	11.69 ± 4.79	114.77 ± 35.84	823.33 ± 313.10	43.17 ± 13.98	55.5 ± 7.03
*t*	−2.208	1.761	−0.994	−1.152	2.290
*P*	*0.030*	*0.081*	*0.322*	*0.252*	*0.024*

**(b):** 

Group	Total cholesterol	Triglyceride	Low-density lipoprotein	High-density lipoprotein
	(mmol/L)	(mmol/L)	(mmol/L)	(mmol/L)
Simvastatin group	4.01 ± 0.94	1.03 ± 0.32	2.47 ± 0.58	1.10 ± 0.20
Control group	4.18 ± 0.85	1.14 ± 0.30	2.60 ± 0.49	1.19 ± 0.29
*t*	−1.057	−1.876	−1.224	−1.906
*P*	*0.293*	*0.063*	*0.224*	*0.059*

^*∗*^ *P* < 0.05 compared with control group. EF: ejection fraction; ICU: intensive care unit.
